# Development and validation of an artificial neural network model for non-invasive gastric cancer screening and diagnosis

**DOI:** 10.1038/s41598-022-26477-4

**Published:** 2022-12-16

**Authors:** Zeyu Fan, Yuxin Guo, Xinrui Gu, Rongrong Huang, Wenjun Miao

**Affiliations:** 1 School of Pharmaceutical Sciences, Nanjing Tech University, Nanjing, 211816 People’s Republic of China; 2 Department of Clinical Laboratory, The Affiliated Drum Tower Hospital, Nanjing University Medical School, Nanjing, 210008 People’s Republic of China; 3 State Key Laboratory of Materials-Oriented Chemical Engineering, Nanjing Tech University, Nanjing, 211816 People’s Republic of China

**Keywords:** Computational biology and bioinformatics, Biomarkers

## Abstract

Non-invasive and cost-effective diagnosis of gastric cancer is essential to improve outcomes. Aim of the study was to establish a neural network model based on patient demographic data and serum biomarker panels to aid gastric cancer diagnosis. A total of 295 patients hospitalized in Nanjing Drum Tower hospital diagnosed with gastric cancer based on tissue biopsy, and 423 healthy volunteers were included in the study. Demographical information and tumor biomarkers were obtained from Hospital Information System (HIS) as original data. Pearson's correlation analysis was applied on 574 individuals’ data (training set, 229 patients and 345 healthy volunteers) to analyze the relationship between each variable and the final diagnostic result. And independent sample *t* test was used to detect the differences of the variables. Finally, a neural network model based on 14 relevant variables was constructed. The model was tested on the validation set (144 individuals including 66 patients and 78 healthy volunteers). The predictive ability of the proposed model was compared with other common machine learning models including logistic regression and random forest. Tumor markers contributing significantly to gastric cancer screening included CA199, CA125, AFP, and CA242 were identified, which might be considered as important inspection items for gastric cancer screening. The accuracy of the model on validation set was 86.8% and the F1-score was 85.0%, which were better than the performance of other models under the same condition. A non-invasive and low-cost artificial neural network model was developed and proved to be a valuable tool to assist gastric cancer diagnosis.

## Introduction

Gastric cancer represents the third leading cause of cancer-related deaths and ranks fifth for cancer incidence worldwide. With over 1 million people diagnosed annually, China accounts for 43.9% of the cancer cases^1,2^. One of the most crucial factors affecting the prognosis of gastric cancer is the cancer stage^3^. The 5-year survival rate of early gastric cancer patients can reach over 90%, wherein, for advanced stage gastric cancer, the rate drops sharply to less than 30%^4,5^. A majority of patients with advanced-stage gastric cancer develop malignant gastric perforation and multiple comorbidities including anemia, infectious disease, bowel obstruction and congestive heart failure^6^, which remains a concern. Despite the fact that early and accurate detection of gastric cancer is of vital importance for the screening, diagnosis and subsequent intervention of gastric cancer patients, due to the scarce and nonspecific symptomatology, gastric cancer appears to be silent in the early stage generally with limited technology to detect.

Currently, excisional biopsy occupies an important position in gastric cancer diagnosis. Subsequent pathologic review offering clinical cancer information such as tumor size, depth of tumor invasion and histologic subtypes are used to direct cancer staging and treatment^7^. However, excisional biopsy is an invasive technique requiring anesthesia and not practicable for repeated sampling over times in order to detect intra-tumor heterogeneity and accomplish routine monitoring. Imaging tests such as endoscopic ultrasound, computed tomography (CT), magnetic resonance imaging (MRI) and positron emission tomography-computed tomography (PET/CT) have also been applied to preoperative staging and evaluation of gastric cancer^8,9^. Although technological innovations in this area have made great contributions to improve the quality of diagnosis, problems such as high cost, low specificity, dependence on the skill of the operator and unavoidable interobserver variability still exist. Biomarker testing is also involved in the diagnosis of gastric cancer^10^, but diagnostic challenge remains as single biomarker detection lacks sensitivity and specificity.

**Figure 1:**
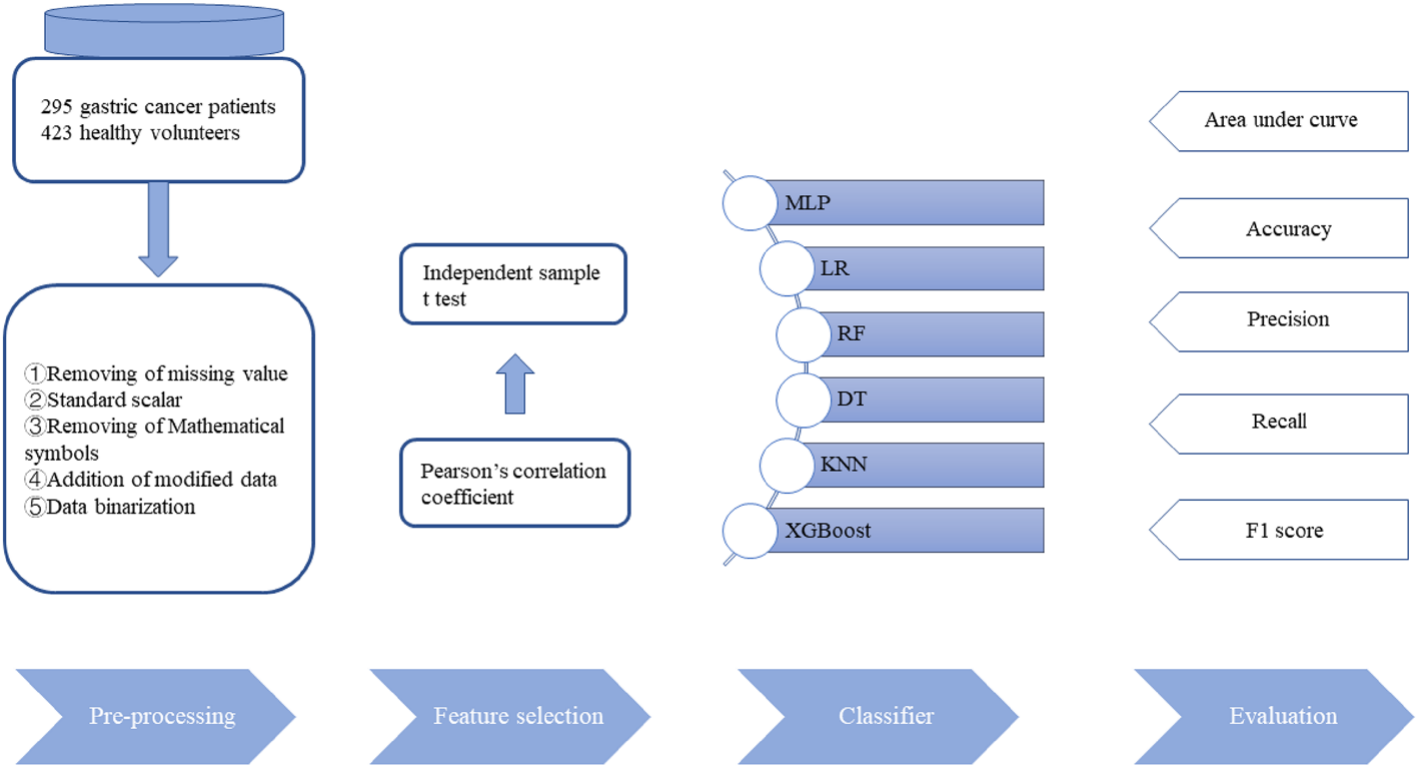
The roadmap of the proposed system based on the CRISP methodology.

Artificial intelligence (AI) with machine learning has drawn great attention in the field of medicine recently^11,12^. Through data mining technology, valuable information can be extracted from large-size medical databases to develop machine learning models for diagnostic and prognostic prediction. The unique characteristics of machine learning including nonlinearity, fault tolerance and can be retrained with update databases are suitable for disease diagnosis. Researches on AI-aided images analysis using machine learning or deep learning has been the mainstream so far^13–15^. For example, Wang and co-workers reported a deep learning framework to predict gastric cancer outcome using resected lymph node (LN) histopathology images^16^. By digitizing hematoxylin–eosin (H&E)-stained LN pathology slides and selecting discovery cohort to train the segmentation network, they proposed a model with excellent accuracy. In particular, they selected a certain number of whole-slide images each year over a period of 6 years to improve the framework robustness and avoid bias. Ba and co-workers further assessed the performance of deep learning assisted gastric cancer diagnosis and proved pathologists with the aid of deep learning accomplished higher detection sensitivity than the ones without (90.63% vs 82.75%)^17^. Adopting AI to enhance endoscopy-based diagnosis is another popular area in this field. Yoon and co-workers reported a lesion-based model for the detection of early gastric cancer^18^. Sakai and co-workers adopted a convolutional neural network-based automatic detection model to enhance the diagnostic capability of endoscopists^19^. Besides pathology^20,21^ and endoscopy, AI has also been employed to assist CT imaging-based diagnosis. Jing and co-workers used deep learning to build a radiomics nomogram to improve lymph node metastasis risk prediction in gastric cancer, which exhibited AUC 0.82 in the test set^22^. In a word, AI techniques are making remarkable progress in assisting gastric cancer diagnosis, however, the clinical data used to construct the above models relies on the excisional biopsy or imaging tests, which are not feasible in routine tests. On the other hand, serum tumor markers detection is cheap and convenient. The drawbacks of tumor markers including low accuracy of single biomarker and the nonlinear relationship between the biomarker and the presence of malignancy can be compensated by taking advantage of machine learning. In this study, an artificial neural network using demographical information and a panel of tumor biomarkers was developed (Fig. 1). A total of 295 gastric cancer patients and 423 healthy volunteers were recruited and divided into training set and validation set. Then an AI-assisted diagnostic system was constructed using multilayer perceptron (MLP) and back propagation. Finally, the diagnostic performance of the model was assessed and compared with other common machine learning models.

## Materials and methods

### Dataset

In this study, 718 samples were contained in the dataset. A total of 295 patients hospitalized in Nanjing Drum Tower hospital from January 2019 to December 2020 diagnosed with gastric cancer based on tissue biopsy were included. Among which 200 were male and the mean patient age was 62.71 (SD = 11.78). 423 healthy volunteers were selected to serve as controls. The mean age was 45.7 (SD = 11.42) and 195 were male. After an initial review of all the clinical parameters, those obtained through invasive or expensive technologies were excluded, along with clinical parameters which have not been reported to be associate with gastric cancer. Following these principles, demographic information (gender, age) and tumor markers (alpha-fetoprotein, carcinoembryonic antigen, etc.) were enrolled in this study and characteristics of the original dataset were summarized in Table 1. This study was approved by the Ethics Committee of Nanjing Drum Tower Hospital, the Affiliated Hospital of Nanjing University Medical School and written informed consent was obtained from participants. All methods were performed in accordance with the relevant guidelines and regulations.

**Table 1:** Sample of the original dataset.

Gender	Age	Inspection result					
		CA242	AFP	CEA	CA724	CA125	CA199
Female	57	8.09	1.40	0.67	12.30	6.00	7.00
Male	43	8.57	< 1.30	2.66	3.17	3.50	11.80
Male	51	7.04	3.10	1.86	< 1.50	9.20	7.49
…	…	…	…	…	…	…	

### Data preprocessing

The original dataset included discrete data and numerical data, in order to standardize the dataset, feature binarization was adopted for the former kind of data, and for the numerical data which could not participate in the calculation directly or exceeded the instrument measurement range, the special characters (greater than or less than symbol) were removed and replaced with modified data. The reason that these original data was not deleted directly was to avoid information loss and reduction of prediction accuracy. All in all, by data modification and binarization, clinical parameters within the normal range from the abnormal ones were differentiated without undermining data integrity, and the result dataset was shown in Table 2.

**Table 2:** Sample of the preprocessed dataset.

Gender	Age	Inspection result	Result state	Inspection result	Result state	…
		CA242	CA242	AFP	AFP	…
0	57	8.09	0	1.40	0	…
1	43	8.57	0	1.30	0	…
1	51	7.04	0	3.10	0	…
…	…	…	…	…	…	…

**Figure 2:**
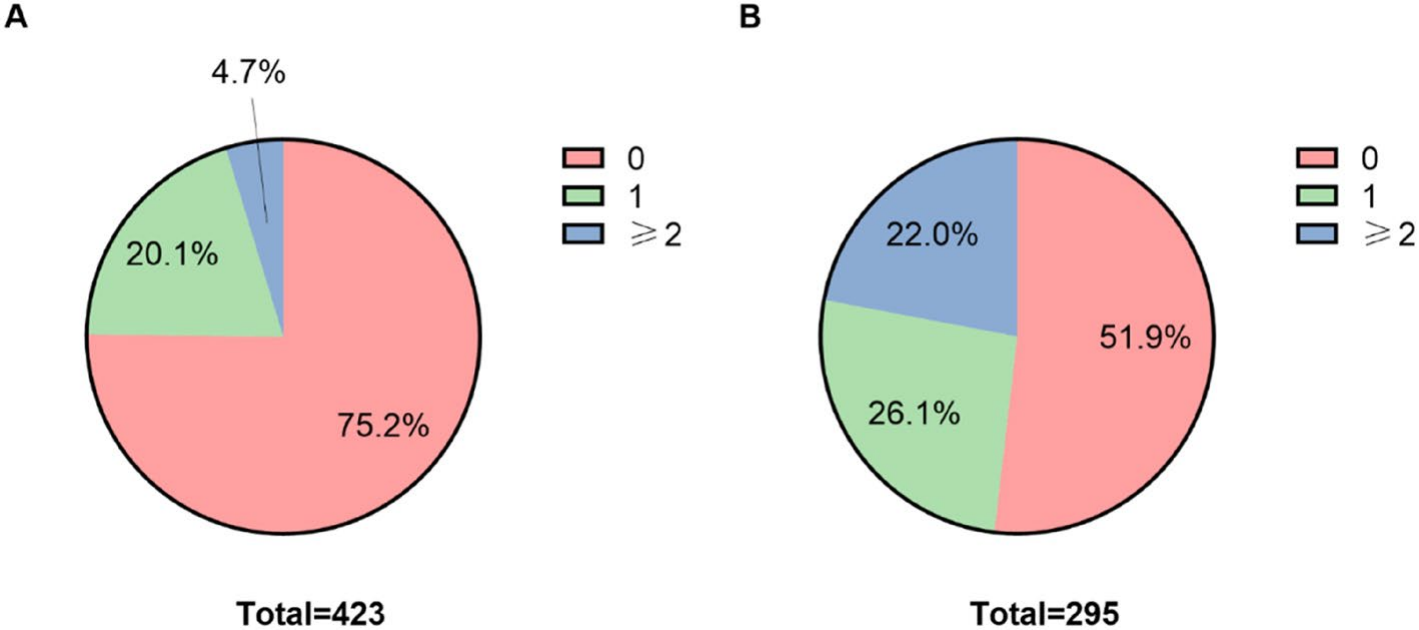
Distribution of the comprehensive index in healthy individuals and gastric cancer patients.

As described above, the original data within the instrument measurement range or below the lower limit of the measurement range was set as 0, and the original data exceeded the upper limit of the measurement range was set as 1 to obtain the modified data, which were then superimposed to obtain a new variable named as the comprehensive index. The dataset was divided into two categories according to whether the individual was diagnosed with gastric cancer or not, and the distribution of the comprehensive index in healthy individuals (n = 423) and patients (n = 295) was shown in Fig. 2.

### Feature extraction

**Figure 3:**
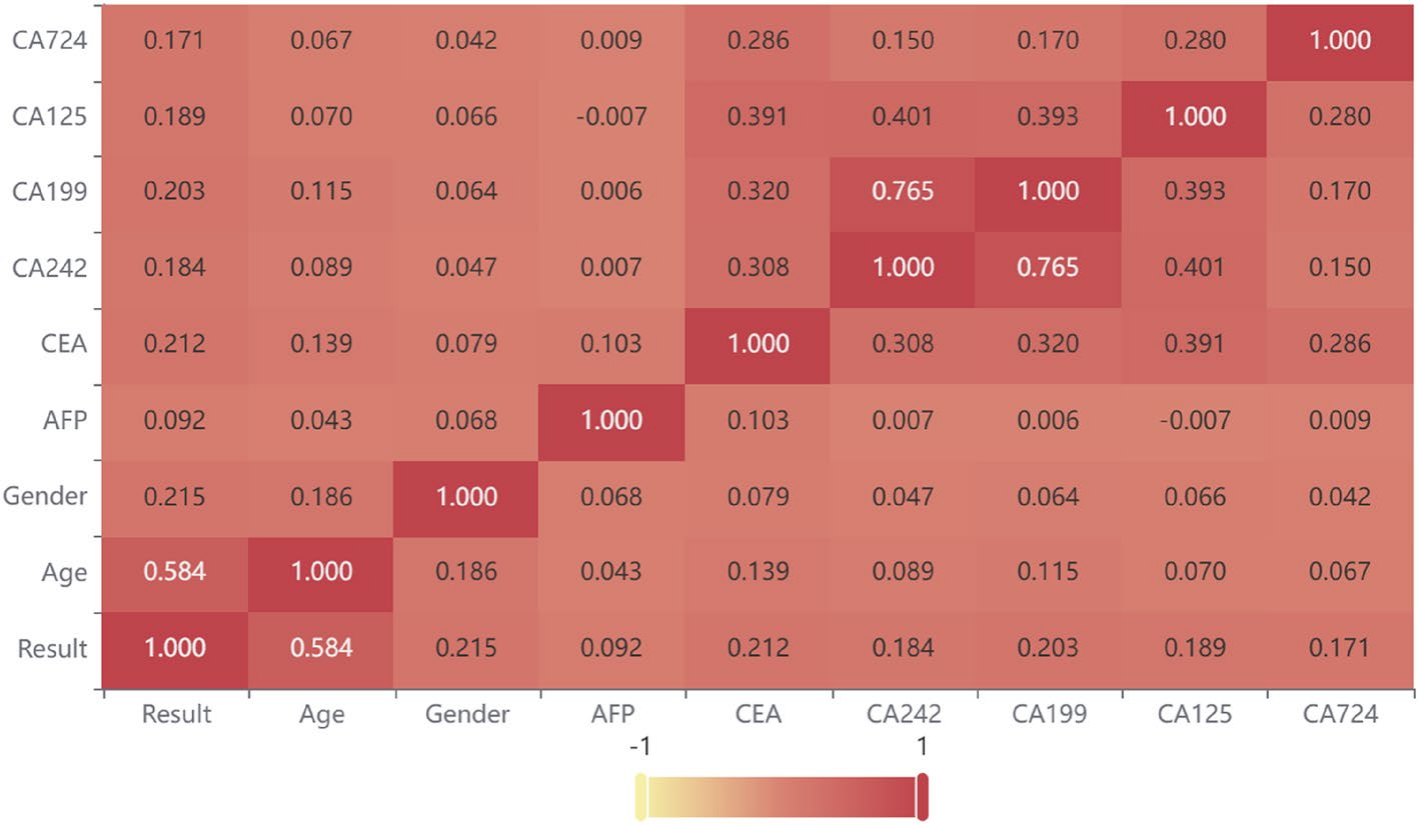
The heatmap showed the Pearson’s correlation coefficient of indicators (the heatmap was created using an online platform for statistical services, SPSSPRO, http://www.spsspro.com).

In order to improve the robustness of the screening model, Pearson bivariate correlation analysis (two-tail) was used to analyze the correlation between each variable and diagnosis results to identify important characteristic variables. Independent sample *t* test was used to evaluate the difference between each variable. As shown in Table 3 and Fig. 3, all the variables selected in this study were significantly correlated with the diagnosis results (*P* < 0.05, inspection result was used in the correlation analysis). The correlation between each variable and the diagnosis results of gastric cancer, significant differences among variables and clinical practicability were the main three factors considered in the selection of modeling variables. According to these principles, the following 14 variables were selected for modeling: gender, age, inspection result (CEA, AFP, CA242, CA125, CA199, CA724), result state (CEA, AFP, CA242, CA125, CA199, CA724).

**Table 3:** Variables significantly correlated with diagnostic results.

Variables	Pearson’s correlation coefficient	*P* values
Gender	0.215	< 0.001
Age	0.584	0.016
CA242	0.184	< 0.001
AFP	0.092	< 0.001
CEA	0.212	< 0.001
CA125	0.189	< 0.001
CA199	0.203	< 0.001
CA724	0.171	< 0.001

### Multilayer perceptron

**Figure 4:**
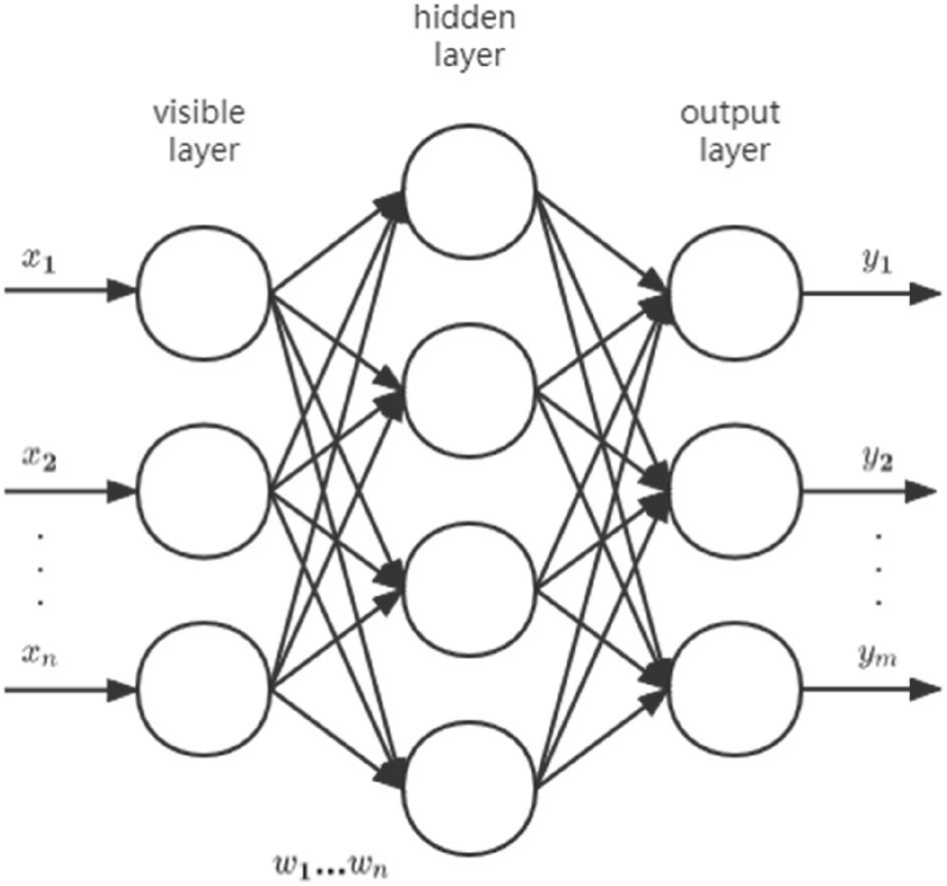
Three layers network structure.

Multilayer perceptron (MLP), also named Artificial Neural Network (ANN), can be divided into three layers: the bottom layer (visible layer), the middle layer (hidden layer), and the last layer (output layer). The layers are fully connected and any neuron in the upper layer is connected to all neurons in the lower layer. Multiple hidden layers can be included in the middle layer. The simplest MLP has only one hidden layer, known as a three-layer structure (Fig. 4).

The visible layer is determined by the input data and n neurons were included in the input n-dimensional vector. Assuming that the visible layer is represented by vector X, then the output of the hidden layer is f(W_1_X + b_1_), in which W_1_ is the weight, b_1_ is the bias, and function f can be commonly used functions (sigmoid function or tanh function). The hidden layer sends data to the output layer can be regarded as a multi-category logistic regression, named softmax regression. The output of the output layer is softmax(W_2_X_1_ + b_2_), in which X_2_ (equal to f(W_1_X + b_1_)) represents the output of the hidden layer. The MLP formula of the above three layers is summarized as follows (Eq. 1):

1 $$\begin{array}{l}f\left(x\right)=G\left({b}^{\left(2\right)}+{W}^{\left(2\right)}\left(s\left({b}^{\left(1\right)}+{W}^{\left(1\right)}x\right)\right)\right)\end{array}$$

The function G is the softmax function mentioned above. Therefore, all the parameters of the MLP represent the connection weights and the bias between the layers, including W1, b1, W2 and b2.

### Model evaluation

The prediction performance of the model was evaluated in terms of confusion matrix, receiver operating characteristic curve (ROC), area under curve (AUC), classification accuracy (ACC), precision, recall and F1-score, etc. using the validation set. Wherein AUC represents the area under the receiver operating characteristic curve, accuracy is defined to be the percentage of correct predictions in the total number of predictions, precision is defined to be the proportion of the predicted positive samples that are actually positive, recall is the percentage of positive samples predicted to be positive and F1-score is the weighted harmonic average of precision and recall.

The prediction performance indexes such as accuracy were calculated based on Table4. The horizontal and vertical coordinates of the receiver operating characteristic curve are represented using False Positive Rate (FPR) and True Positive Rate (TPR) respectively (Eqs. 2 and 3).

**Table 4:** Illustration of confusion matrices.

Reality	Prediction	
	Positive	Negative
Positive	TP (True Positive)	FN (False Negative)
Negative	FP (False Positive)	TN (True Negative)

2 $$\begin{array}{c}FPR= \frac{FP}{FP+TN}\#\end{array}$$

3 $$\begin{array}{c}TPR=Recall= \frac{TP}{TP+FN}\#\end{array}$$

Other evaluation indexes are calculated as follows (Eqs. 4, 5 and 6).

4 $$\begin{array}{c}Accuracy=\frac{TP+TN}{TP+TN+FP+FN}\end{array}$$

5 $$\begin{array}{c}Precision=\frac{TP}{TP+FP}\end{array}$$

6 $$\begin{array}{c}{F}_{1}=2*\frac{Precision*Recall}{Precision+Recall}\end{array}$$

### Model comparison

The performance of the neural network model was compared with other common machine learning models including logistic regression, *k*-Nearest Neighbor, decision tree, random forest and XGBoost. The model creation and evaluation were performed using JetBrains PyCharm 2018.3 software, and the correlation analysis and *t*-test were performed using IBM SPSS Statistics 26 software.

## Results

### Performance assessment of neural network model

**Figure 5:**
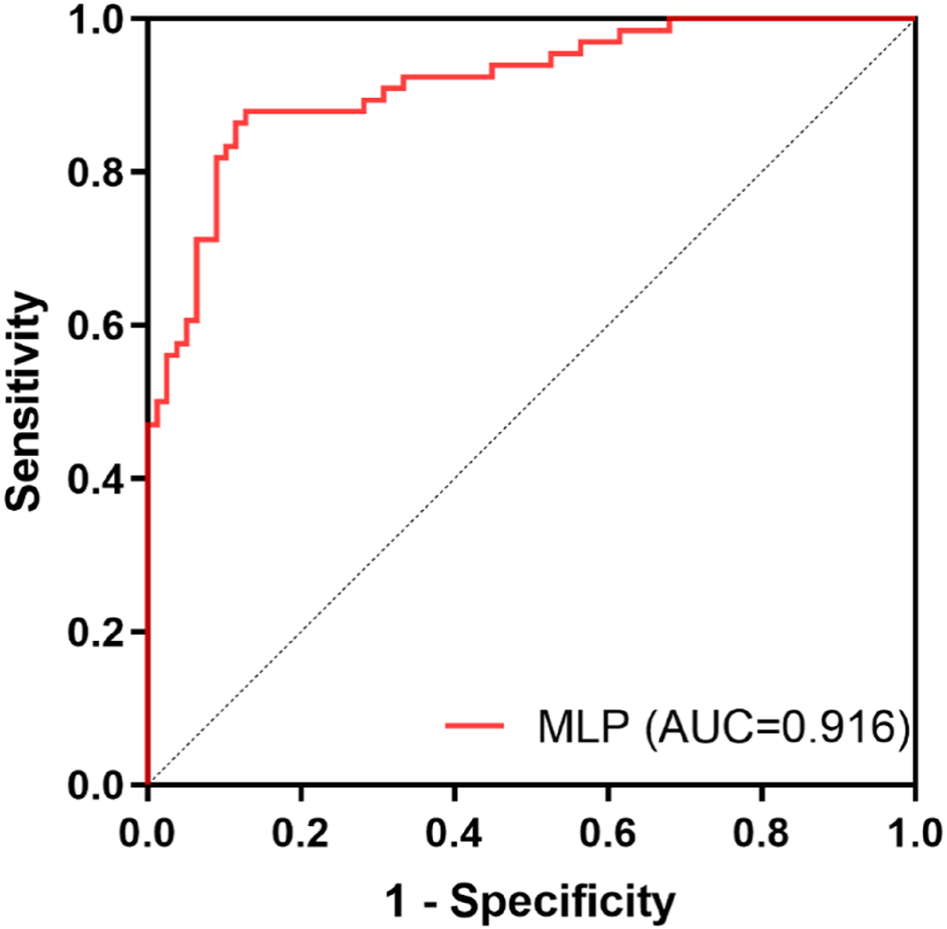
ROC curve of the neural network model (MLP).

In this work, the final result was set as the target variable, and the preprocessed variable was set as the decision variable. Due to the large amount of data involved in this study, “adam” was selected to serve as the solver for weight optimization, and “ReLU” was selected to serve as the activation function of hidden layer. Before training, the number of the hidden layers for the ANN was set to 3 and the number of neurons for each hidden layer is (64, 32, 32). Besides, maximum number of iterations (1000), L2 penalty parameter (1e−3) and default values for all the other parameters were used to construct the neural network model. The neural network model was trained using the training set (574 specimens) and the performance of the model was valuated using the validation set (144 specimens). As shown in Fig. 5, the area under the curve was 0.916 [*P* < 0.001, 95% confidence interval (CI): 0.870–0.962]. And the TPR and FPR corresponding to thresholds ranging from 0.25 to 0.51 were presented in Table 5.

**Table 5:** The TPR and FPR corresponding to thresholds ranging from 0.25 to 0.51.

Thresholds	TPR	FPR
0.510	0.818	0.090
0.477	0.818	0.103
0.469	0.833	0.103
0.462	0.833	0.115
0.418	0.864	0.115
0.406	0.864	0.128
0.398	0.879	0.128
0.275	0.879	0.282
0.255	0.894	0.282

**Figure 6:**
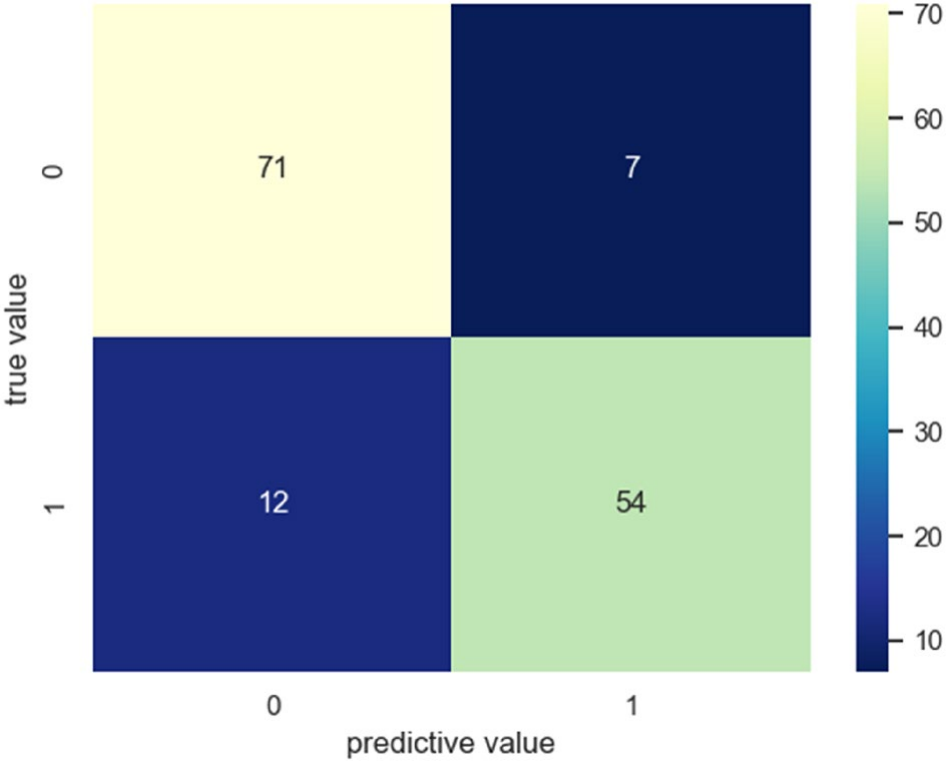
Confusion matrix of the classification model when the threshold was 0.5. Negative samples were represented by 0 and positive samples were represented by 1.

Figure 6 showed the confusion matrix of the model when the cutoff probability value was 0.5, and the color scale described the grid color corresponding to the occurrence times of each condition. The confusion matrix showed that out of 144 test results, 54 positive samples were predicted to be positive, 12 positive samples were predicted to be negative, 71 negative samples were predicted to be negative, and 7 negative samples were predicted to be positive. The accuracy of the model on the validation set was 86.8%, the precision rate was 88.5%, the recall rate was 81.8% and the F1-score was 85.0%.

### Variable importance

The PI (Permutation importance) method was applied to the trained neural network model to obtain the importance coefficients of each variable. By adding the importance coefficients of inspection result and result status, the variable corresponding to each tumor marker was obtained (Table 6).

**Table 6:** Importance of variables in the proposed gastric cancer screening model.

Order of importance	Variables	Importance
1	Age	0.325
2	CA199	0.180
3	CA125	0.148
4	AFP	0.112
5	CA242	0.096
6	CEA	0.059
7	CA724	0.047
8	Gender	0.034

### Model comparison

**Figure 7:**
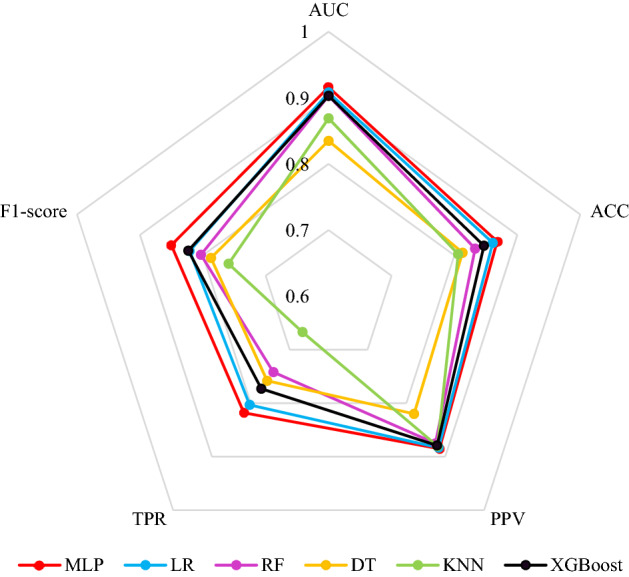
Comparison of each model when cut-off was 0.5. MLP (neural network model), LR (Logistic Regression), RF (Random Forest), DT (Decision Tree), KNN (*k*-Nearest Neighbor), XGBoost (eXtreme gradient boosting), AUC (area under the receiver operating characteristic curve), ACC (accuracy), PPV (precision, positive predictive value), TPR (recall, true positive rate).

Using the same training set and validation set as the neural network model, five other common classification models (Logistic Regression, K-Nearest Neighbor, Decision Tree, Random Forest, eXtreme gradient boosting) were constructed. The performance of each model was evaluated by five indexes: area under the curve, accuracy, precision, recall and F1 score (Fig. 7, Table S1).

Results showed that MLP model outperformed the other models in all five evaluation indexes (AUC, accuracy, precision, recall and F1 score), and the paired sample *t*-test showed that there were significant differences between the indexes of the MLP and the average level of the five models (*P* < 0.05), indicating that the proposed neural network model exhibited higher predictive ability in gastric cancer screening than the other commonly used models.

## Discussion

This study retrospectively analyzed the data of 295 gastric cancer patients and 423 healthy volunteers to develop an intelligent model for assisting gastric cancer diagnosis with non-invasive and low-cost items. Firstly, the dataset was preprocessed and the modified data was obtained at the same time. Then, the correlation of variables was checked using Pearson’s correlation coefficient to exclude irrelevant variables. The dataset with full and selected variables was fed into the ML models separately. Finally, the models’ performance was evaluated and compared based on the confusion matrix criteria including the five evaluation indexes. The results of comparing the six selected ML algorithms after feature selection showed that the MLP achieved the highest performance in the diagnosis of gastric cancer patients with an accuracy of 86.8%, precision of 88.5%, recall of 81.8%, F1-score of 85.5%, AUC of 91.6%. Algorithms close to this level were LR and XGBoost, with AUC of 90.8% and 90.3%, respectively. However, other evaluation indexes of these two algorithms were significantly worse than those of MLP.

### Evaluation of the modified data

In the data preprocessing stage, modified data were introduced to complement the original numerical data which were not able to participate in the operation directly with elimination of the error caused by removal of the special characters (greater than or less than symbol). Using paired sample *t* test, significant differences between the two groups of data in the comprehensive index (*P* < 0.05) were identified, indicating that the comprehensive index was strongly related to gastric cancer. Meanwhile, the accuracy of the trained model was down to 0.806 without the modified data. The F1-score was 0.768, which was 9.6% lower than after the usage of modified data. And the area under curve also decreased slightly. All these results proved the importance of the modified data we introduced.

### Selection of model threshold in clinical application

In this study, default cut-off value (0.5) was frequently used as this point is the closest to the expected value of the upper-left corner (0, 1) in the ROC curve, and both sensitivity and specificity reach a high level. Nevertheless, in clinical practice, misdiagnosis of positive patients is more serious than misdiagnosis of negative healthy people. Therefore, the cut-off should be reduced appropriately to transfer the model into clinical application, which means certain specificity should be sacrificed within the acceptable range to improve the sensitivity as much as possible. The thresholds ranging from 0.25 to 0.51 in the ROC curve and their corresponding TPR and FPR were shown in Table 5 According to the principle described above, the priority of TPR should be slightly higher than FPR, and the threshold values between 0.398 to 0.406 could be selected as the cut-off values as outside this interval might result in negative impact on the comprehensive performance of the model due to the sudden decline of TPR or sudden rise of FPR.

### Variable importance analysis

From the perspective of the demographic information, age occupied the first place in the importance of variables with a coefficient of 0.325 and played a very important role in the model, meanwhile, gender ranked the bottom with an importance coefficient of 0.034 and barely participate in decision-making. According to the general distribution of age and gender of gastric cancer in China, the majority of gastric cancer patients belong to middle-aged and elderly. The incidence of gastric cancer is relatively low in those under 35 years old. And the incidence of male is higher than female, with a ratio of 2:1, approximately^23^. These statistics were consistent with the sample we selected. At the same time, the age indicator in the model was roughly in line with the actual situation. However, age is not the determining factor of gastric cancer, nor could age be the most important factor in gastric cancer decision-making. Although age was strongly related to gastric cancer according to the analysis of the data, whether it should be used in the prediction model and how does age impact on gastric cancer decision-making still needs further discussion. In addition, there was no obvious difference in the incidence of gastric cancer between male and female, which may be related to the interaction between gender and age in the incidence of gastric cancer, or gender might be an indecisive factor in the diagnosis of gastric cancer compared with other tumor markers, resulting in the low importance coefficient of gender.

From the perspective of tumor markers, the importance coefficient of CA199, CA125 and AFP were high, and the coefficient difference between these variables was relatively small, indicating that these variables played important roles in gastric cancer screening. However, single biomarker detection lacks sensitivity and specificity, it is more valuable in clinical practice to performed combined detection. In addition, CA199 ranked second among all variables and first among tumor markers with an importance coefficient of 0.180, indicating that CA199 might have a non-negligible importance in the diagnosis of gastric cancer, which is consistent with the research results of Yang Rong et al.^24^. In this study, the participation of CA724 in the model was very low, so we speculated that the value of CA724 in gastric cancer screening was dispensable, which was consistent with the results of Wang et al.^25^. Besides, in the previous study on the correlation of indicators, we found that the Pearson’s correlation coefficient between CA242 and CA199 reached 0.765, which was a strong correlation. Even though there is no relevant literature reported at present and the functionality of the correlation between CA242 and CA199 is unclear, the phenomenon represents a putative biomarker candidate for gastric cancer diagnosis and could be a potential topic for further clinical research.

### Limitations and implications

The proposed model is likely to predict the patients with gastric cancer accurately based on patient demographic data and serum biomarker panels, which makes it applicable to clinical practice. However, potential limitations still exist. First, we assumed that the results of diagnosis were correct for all samples involved in this study and the measurement of the indicators was rigorous. Then, we introduced new modified data to describe the values that fell outside the defined range, which undoubtedly expanded the dimension of data and increased the amount of calculation. This was applicable under the conditions of our study, but not in the case of high-throughput datasets. Second, we dealt with a single-center dataset with a limited sample size that might affect the quality of modeling, comprehensiveness, and generalizability of data. Third, the dataset did not collect data on Helicobacter pylori, pepsinogen, smoking or other non-invasive factors that may lead to gastric cancer. The inclusion of these factors may increase the predictive power of the models. Furthermore, there was still a certain gap between the predictive power of this non-invasive approach and the current mainstream recognition model based on machine learning for gastric cancer slides. Therefore, it is recommended that more studies be conducted after more accurate validations to improve the quality of modeling and minimize prognosis bias.

## Conclusion

Serum tumor markers can be detected through convenient, non-invasive and low-cost clinical routine test. However, challenge remains as single biomarker detection lacks accuracy, and statistical model is lacking for the data analysis of combined detection. Here we established a neural network model based on patient demographic data and serum biomarker panels to aid gastric cancer screening and diagnosis. The AUC of the model reached a relatively high level (0.916) compared with other commonly used models. In addition, clinical parameters contributing significantly to gastric cancer screening included age (which needs further discussion), CA199, CA125, AFP, and CA242 were identified and might be considered as important inspection items for gastric cancer screening. Limited by the low dimension of sample data, other biomarkers related to gastric cancer such as gastrin, MG7-Ag (gastric carcinoma-associated MG7-Ag) were not included in this study. Despite the big gap in specificity between the proposed model and mainstream machine-assisted pathological image recognition, it is still a valuable tool to assist physicians in large-scale initial screening. In summary, using clinical data and multi-layer perceptron, a gastric cancer screening model with good predictive performance was constructed to assist physicians in gastric cancer diagnosis. Furthermore, with the ability to improve the diagnostic value of laboratory tests results, the proposed model can be trained to be applied in various diseases other than gastric cancer.

## Supplementary Information

Supplementary Table S1.

## Data Availability

The datasets used and/or analyzed during the current study available from the corresponding author on reasonable request.
