# Supplementary material for: Development and validation of an artificial neural network model for non-invasive gastric cancer screening and diagnosis
Source: Sci Rep. 2022 Dec 16;12:21795. doi: 10.1038/s41598-022-26477-4 (PMC9758153; doi:10.1038/s41598-022-26477-4)
Supplement: Supplementary file 1 — Supplementary Table S1. [file 41598_2022_26477_MOESM1_ESM.docx]

**Supplementary information for**

**Development and validation of an artificial neural network model for**

**non-invasive gastric cancer screening and diagnosis**

Zeyu Fan ^a^, Yuxin Guo ^a^, Xinrui Gu ^b^, Rongrong Huang ^a,*^, Wenjun Miao ^a, c,*^

^a^ School of Pharmaceutical Sciences, Nanjing Tech University, Nanjing 211816, P. R. China

^b^ Department of Clinical Laboratory, the Affiliated Drum Tower Hospital of Nanjing University Medical School, Nanjing 210008, P. R. China

^c^ State Key Laboratory of Materials-Oriented Chemical Engineering, Nanjing Tech University, Nanjing 211816, P. R. China

E-mail: [miaowj@njtech.edu.cn](mailto:miaowj@njtech.edu.cn) (W. Miao), [huangrongrong@njtech.edu.cn](mailto:huangrongrong@njtech.edu.cn) (R. Huang)

**Table S1.** Details of evaluation indexes of each algorithm on the same dataset.

| **Classifiers** | **MLP** | **LR** | **RF** | **DT** | **KNN** | **XGBoost** |
| --- | --- | --- | --- | --- | --- | --- |
| **AUC** | 0.916 | 0.908 | 0.903 | 0.835 | 0.869 | 0.903 |
| **Accuracy** | 0.868 | 0.861 | 0.833 | 0.813 | 0.806 | 0.847 |
| **Precision** | 0.885 | 0.883 | 0.875 | 0.820 | 0.880 | 0.879 |
| **Recall** | 0.818 | 0.803 | 0.742 | 0.758 | 0.667 | 0.773 |
| **F1-score** | 0.850 | 0.821 | 0.803 | 0.787 | 0.759 | 0.823 |
